# Multiomic prioritisation of risk genes for anorexia nervosa

**DOI:** 10.1017/S0033291723000235

**Published:** 2023-10

**Authors:** Danielle M. Adams, William R. Reay, Murray J. Cairns

**Affiliations:** 1 School of Biomedical Sciences and Pharmacy, Centre for Complex Disease Neurobiology and Precision Medicine, The University of Newcastle, Callaghan, NSW, Australia; 2 Precision Medicine Research Program, Hunter Medical Research Institute, Newcastle, NSW, Australia

**Keywords:** Alternative splicing, anorexia nervosa, mRNA, protein expression, PWAS, TWAS

## Abstract

**Background:**

Anorexia nervosa (AN) is a psychiatric disorder associated with marked morbidity. Whilst AN genetic studies could identify novel treatment targets, integration of functional genomics data, including transcriptomics and proteomics, would assist to disentangle correlated signals and reveal causally associated genes.

**Methods:**

We used models of genetically imputed expression and splicing from 14 tissues, leveraging mRNA, protein, and mRNA alternative splicing weights to identify genes, proteins, and transcripts, respectively, associated with AN risk. This was accomplished through transcriptome, proteome, and spliceosome-wide association studies, followed by conditional analysis and finemapping to prioritise candidate causal genes.

**Results:**

We uncovered 134 genes for which genetically predicted mRNA expression was associated with AN after multiple-testing correction, as well as four proteins and 16 alternatively spliced transcripts. Conditional analysis of these significantly associated genes on other proximal association signals resulted in 97 genes independently associated with AN. Moreover, probabilistic finemapping further refined these associations and prioritised putative causal genes. The gene *WDR6*, for which increased genetically predicted mRNA expression was correlated with AN, was strongly supported by both conditional analyses and finemapping. Pathway analysis of genes revealed by finemapping identified the pathway *regulation of immune system process* (overlapping genes = *MST1*, *TREX1*, *PRKAR2A*, *PROS1*) as statistically overrepresented.

**Conclusions:**

We leveraged multiomic datasets to genetically prioritise novel risk genes for AN. Multiple-lines of evidence support that *WDR6* is associated with AN, whilst other prioritised genes were enriched within immune related pathways, further supporting the role of the immune system in AN.

## Introduction

Anorexia nervosa (AN) is a complex psychiatric disorder associated with alterations to satiety, activity, and self-perception that results in severe mental distress and malnourishment (Sibeoni et al., 2017). Cognitive behavioural therapy and weight rehabilitation are the first-line treatments for AN, as there are still no approved pharmacotherapies for the disorder (Wonderlich, Bulik, Schmidt, Steiger, & Hoek, 2020). Unravelling the biological complexity of AN onset and its clinical course will be key to developing more effective interventions and improving clinical management.

AN is influenced by genetic and environmental factors, with twin studies estimating heritability at 56% (Bulik et al., 2006), and common variants are now shown to account for around 10–20% of liability scale heritability through genome-wide association studies (GWAS) (Hirtz & Hinney, 2020). The most recent AN GWAS uncovered eight independent genome-wide associated loci (Watson et al., 2019). GWAS present an opportunity to better understand the biology of AN, as well as potentially identify novel treatment targets and opportunities for drug repurposing (Reay & Cairns, 2021). For example, the latest AN GWAS consolidated the strong genetic overlap between the disorder and systemic metabolic factors like cholesterol and insulin, leading to AN being conceptualised a ‘metabo-psychiatric’ disorder (Adams, Reay, Geaghan, & Cairns, 2021; Watson et al., 2019). An ongoing challenge in the field of AN genetics is to identify key genes and biological systems that are informative to the pathogenesis of the disorder and may be relevant for treatment.

One way to approach this challenge is through gene-based aggregation methods that can increase power to detect associations beyond genome-wide significant loci and yield more biologically relevant information. In other words, rather than studying individual risk variants, these data can be collapsed at the level of genes to reduce multiple-testing burden and resolve key disorder-associated biological processes. For example, transcriptome wide association studies (TWAS) achieve this by integrating mRNA expression data with GWAS association data to detect genes for which genetically predicted expression is associated with the trait (Wainberg et al., 2019). TWAS can be conceptualised as a genetic approach to more traditional differential expression analyses. Specifically, rather than directly measuring mRNA expression in cases and controls, estimates of genetic effects on mRNA expression are integrated with the effect of those same genetic variants on a phenotype or disorder of interest. The expression component of this approach constructs a model that predicts mRNA expression for each gene using genetic variants. As a result, TWAS is capable of prioritising genes that may be involved in the disorder and assign them a direction of genetically predicted expression that is odds increasing (Wainberg et al., 2019). The direction of effect associated with the disorder derived from TWAS, that is, upregulation or downregulation, is particularly useful in the context of identifying compounds that could reverse the risk-increasing direction of expression. This method can also be extended to other quantitative functional data such as protein and alternatively spliced mRNA isoform abundance. Despite these useful features, TWAS alone is not a test of causality as genes identified may arise due to the confounding factors like co-regulation between genes or linkage disequilibrium between the variants associated with expression (Reay & Cairns, 2021). However, TWAS can be subjected to different statistical approaches to attempt to increase the fidelity to identify true risk genes for a trait (Hall et al., 2019; Mancuso et al., 2019).

AN TWAS have previously been performed and revealed some insights into the pathogenesis of the disorder (Chatzinakos et al., 2020; Cheng et al., 2020; Johnson et al., 2022). For example, TWAS using the S-PrediXcan approach (Barbeira et al., 2018), was previously performed in the study outlining the largest AN GWAS, uncovering 36 genes with predicted expression associated with the disorder using data from Genotype-Tissue Expression (GTEx) project brain and blood data (Watson et al., 2019). However, these studies in AN have only considered mRNA expression, which may miss effects mediated from alternative splicing or protein expression. Moreover, large sample size post-mortem brain datasets like the PsychENCODE consortium (*N*_Samples_ > 1000) with expression and genetic data available present an opportunity to boost power to discover AN associated genes through TWAS. In this study, we utilise models of genetically predicted mRNA expression, protein expression, and alternative mRNA isoform abundance to prioritise genes involved in AN. These association signals were further refined through conditional analysis and finemapping to reveal several genes including *WDR6* that may play a role in AN biology. Pathways analysis also implicated regulation of *immune system process* with gene set functional analysis including signal from *MST1*, *TREX1*, *PRKAR2A* and *PROS1*.

## Materials and methods

### Overview of study

We aimed to prioritise genes associated with AN and implicate potential mechanisms related to expression and/or splicing. Firstly, we conducted a comprehensive brain and blood-based transcriptome-wide association study (TWAS) (Gamazon et al., 2015; Gusev et al., 2016; Reay & Cairns, 2021). TWAS requires expression data which can be imputed from independent SNP-mRNA expression weights from multivariate models of *cis*-acting genetically regulated expression (GReX). TWAS then compares imputed expression with SNP-AN effect sizes to test the association between predicted expression and the odds of AN. Genes uncovered from this approach that survived multiple-testing correction were then further probed to refine candidate causal genes through conditional analysis and probabilistic finemapping (Gusev et al., 2016; Mancuso et al., 2019). Whilst mRNA expression is arguably the most well studied cellular readout, genes may operate more specifically in the pathogenesis of AN through dysregulation of other factors like protein expression and alternative splicing. As a result, we leveraged SNP weights, where available, for protein expression and splicing to also perform a proteome-wide association study (PWAS) and an alternative splicing based test (spliceWAS). Notably, PWAS and spliceWAS analyses have not previously be published for AN. Genes were prioritised based on evidence from mRNA, protein, and alternative isoform expression and then subjected to further *in silico* analyses related to overrepresentation in biological pathways. The AN GWAS utilised in this study was a meta-analysis encompassing European ancestry cohorts that totalled 16 992 cases and 55 525 controls. In the AN GWAS, case status was mostly ascertained from online questionnaires or structured interviews based on standardised clinical criteria, for example, DSM-IV, whilst the UK Biobank derived cases were self-reported. Further details related to collection of samples, phenotype acquisition, and GWAS approach are described in the original publication (Watson et al., 2019).

### Weights for genetically predicted mRNA, protein, and splicing

Brain related SNP weights (multivariate GReX) for TWAS were derived from GTEx v7 and PsychENCODE, whilst whole blood weights were also obtained from GTEx v7 (Gandal et al., 2018; Gusev et al., 2016). The GTEx v7 SNP weights comprise data from twelve different brain regions – with the sample sizes of the cohorts utilised for GReX estimation as follows: amygdala (*N* = 88), anterior cingulate cortex (*N* = 109), caudate (*N* = 144), cerebellar hemisphere (*N* = 125), cerebellum (*N* = 154), cortex (*N* = 136), frontal cortex (*N* = 136), hippocampus (*N* = 111), hypothalamus (*N* = 108), nucleus accumbens (*N* = 130), putamen (*N* = 80) and substantia nigra (*N* = 80). HapMap3 SNPs from the 1000 genomes phase 3 European reference panel were used as a linkage disequilibrium (LD) estimate, to correspond with how the weights were calculated with those same HapMap3 SNPs. The TWAS using whole blood weights utilised the same LD reference strategy, with 369 GTEx v7 participants in these models. Frontal or cerebral cortex tissue from the larger PsychENCODE cohort was also utilised in terms of SNP weights, with a sample size of 1695. As described in the original PsychENCODE publication, gene-wise GReX were estimated for all imputed SNPs, not just the HapMap3 panel, and thus, we utilised the full suite of the phase 3 1000 genomes European subset as the LD reference. There were two tissues for which SNP weights related to protein expression were available – the dorsolateral prefrontal cortex (DLPFC, *N* = 376) and plasma (*N* = 7213) (Wingo et al., 2021; Zhang et al., 2021). It should be noted that the plasma weights were derived from the European subset of the study cohort and the authors only used the elastic net method to derive GReX. Analogous to the difference between the GTEx v7 and PsychENCODE studies above, the DLPFC SNP weights were estimated using the HapMap3 panel, and thus, we only used those SNPs as an LD reference. The plasma PWAS utilised the full reference panel. Finally, the splicing related weights were derived from the DLPFC samples from the Common Mind Consortium (*N* = 452) which utilised the HapMap3 restricted 1000 genomes panel (Gusev et al., 2018).

### Implementation of the FUSION pipeline

We conducted TWAS/PWAS/spliceWAS using the FUSION package (Gusev et al., 2016). Specifically, the *FUSION.assoc_test.R* script was utilised, with the best performing GReX model selected from five-fold cross-validation (*R*^2^) and the SNP weight set selected by FUSION for calculating the TWAS *Z* score. In accordance with usual practice for the FUSION approach, only genes/transcripts with significantly non-zero *cis*-acting heritability (*cis*-*h*^2^) are included. Prior to analysis, summary statistics were also munged whereby SNPs were retained with an imputation INFO > 0.9, as well as removing indels, strand ambiguous SNPs and SNPs with MAF < 0.01. We corrected for the number of non-missing models tested for the TWAS, PWAS, and spliceWAS independently. Our primary method for correcting for multiple testing was the Bonferroni approach considering all models tested for each modality (TWAS, PWAS, and spliceWAS independently), however, this is inherently conservative due to genes having a GReX model in multiple tissues and correlations between genes. As a result, we also used a more exploratory Benjamini-Hochberg false discovery rate (FDR) approach for the TWAS, PWAS, and spliceWAS separately.

### Conditional analyses and probabilistic finemapping

We applied conditional analysis via the FUSION framework (*FUSION.post_process.R*) to the regions of significant genes after correction to investigate the proportion of implicated genes in any given locus that are independently associated. Specifically, jointly significant genes retain their significance after jointly estimating association for all models within a 500 000 base pair region of a significant gene. Marginally associated genes, which are not jointly significant, likely arise due to factors such as genes for which predicted expression was correlated.

Moreover, we applied probabilistic finemapping to prioritise candidate causal genes from any locus in the AN GWAS summary statistics with at least a suggestively significant SNP (*p* < 1 × 10^−5^) using the FOCUS method (Mancuso et al., 2019). The default prior (*p* = 1 × 10^−3^), and prior variance (*nσ*^2^ = 40), were utilised to approximate Bayes' factors such that the posterior inclusion probability (*PIP*) of each gene being a member of a credible set with 90% probability of containing the causal gene could be derived. Finemapping was performed with default tissue prioritisation, as well as the prioritisation of brain tissue. In the TWAS, there were two reference panels utilised for finemapping – for genes uncovered from a GTEx v7 tissue, we utilised the default combined FOCUS SNP weight set which collated GTEx v7 tissues, DLPFC (CommonMind), blood (YFS, NTR), and adipose (METSIM) SNP weight sets (https://www.dropbox.com/s/ep3dzlqnp7p8e5j/focus.db?dl=0), with genes discovered using the PsychENCODE weights finemapped specifically using that panel given the different LD parameters and its more complete set of genes with *cis-*heritable models in that one tissue. The multi-tissue finemapping panel contains several other non-brain tissues, and thus, some GReX models that would not have been available in brain and blood. We sought to balance maximising the number of models available for finemapping, whilst acknowledging that some of the tissues in this panel are less likely to be disease relevant. As FOCUS allows the null mode that the causal feature is not typed to be predicted as a possible member of the credible set, we excluded any genes for which that occurred. The credible set was defined by summing normalised *PIP* such that *ρ* was exceeded, sorting the genes, and then including those genes until at least *ρ* of the normalised-posterior mass is explained, as described in more detail elsewhere (Mancuso et al., 2019; Reay et al., 2021). As an exploratory analysis, we also applied finemapping to the PWAS and spliceWAS results, although the limited number of SNP weights available for protein and alternative splicing means that the probability of a causal gene for any region not being present is higher.

### Investigation of prioritised AN associated genes

We investigated two sets of prioritised genes *in silico*: (1) conditionally independent associations (TWAS/PWAS/spliceWAS) from marginally significant signals (FDR < 0.05) that are at least nominally jointly significant (*p* < 0.05), and (2) genes in the finemapped 90% credible set with *PIP* > 0.4 and the absence of the null model in the credible set. Firstly, we considered biological pathways and other ontological sets for which these two sets of genes could separately be overrepresented via the g:Profiler framework and the Benjamini-Hochberg method for multiple-testing correction (Reimand, Kull, Peterson, Hansen, & Vilo, 2007). We used the default background for gene-set enrichment (statistical domain size) in g:Profiler of only genes annotated to at one least domain out of the thousands of gene-sets considered.

## Results

### Novel AN risk genes uncovered through genetically predicted expression or splicing

**Fig. 1.**
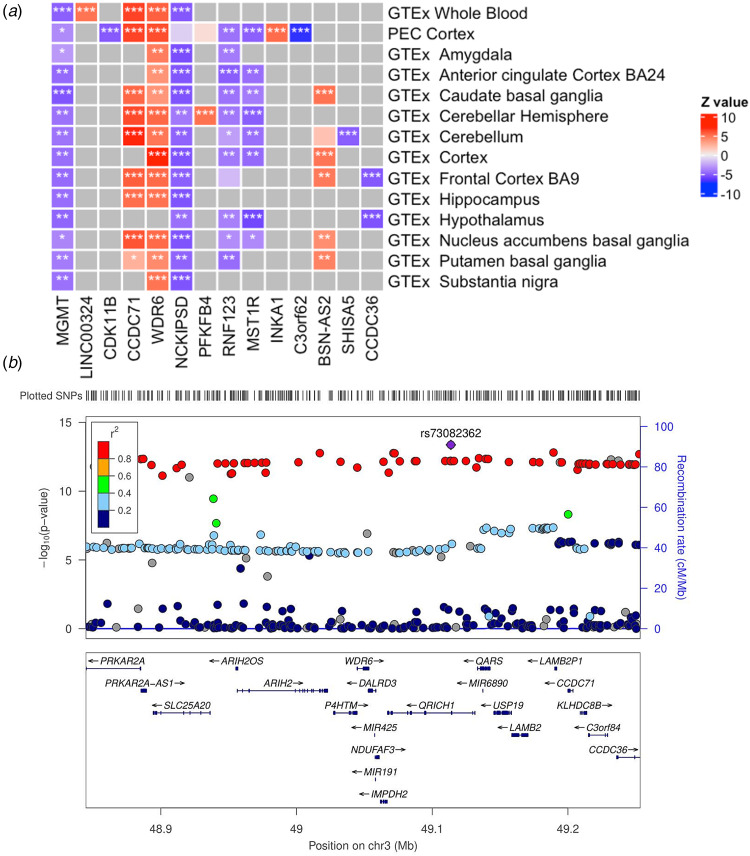
TWAS associations and region plot of the densely associated AN signal on chromosome 3. a: Heatmap of genes with at least one Bonferroni significant eQTL tissue associated with AN. Red indicates positive *z* scores; blue indicates negative *z* scores (legend). Columns indicates genes, rows indicate tissue models. * Indicates nominally significant genes, ** indicates Benjamini-Hochberg significant associations, *** indicates Bonferroni significant associations. Grey squares indicate that a significantly cis-heritable model of imputed expression data was unavailable in that tissue. b: Relative AN gene and SNP locations and significance. Points in the top panel indicate SNPs, legend indicates *r*^2^, left side *y*-axis indicates the negative log transformed *p* value of SNPs, right side *y*-axis indicates the recombination rate (cM/Mb). The bottom panel indicates the location of genes relative to the top panel SNPs. Plot generated using ZoomLocus (Pruim et al., 2010) with 200 kb flanking size. The SNP with the most significant *p* value in this region: rs73082362 is highlighted.

Firstly, a TWAS was performed using models of genetically predicted mRNA expression across twelve brain regions from GTEx, cortical samples from the PsychENCODE consortium, and whole blood GTEx samples. This was the most well-powered approach as there were 57 596 mRNA models, that is genetically predicted models of expression, available to test, with the number of unique genes totalling 11242, 12183, and 5915, for GTEx brain, PsychENCODE brain, and GTEx whole blood samples, respectively (online Supplementary Table S1). TWAS tested the association between genetically predicted expression of these mRNA and the odds of AN. Correcting for all 57 596 genetic models of mRNA expression (*p* < 8.68 × 10^−7^) revealed 40 association signals (14 unique genes), with several genes revealed in multiple brain regions (online Supplementary Table S1). We note here that ‘signals’ refers to any detected association in a tissue, which is different from unique genes as genes often have GReX models available to test in multiple tissues. The most significant association signals were found in a gene dense region on chromosome 3 (Fig. 1), in line with expectation given the significant GWAS signal for AN (chromosome 3: 47 588 253–51 368 253) overlaps this cluster of significant genes. Upregulation of the gene encoding WD Repeat Domain 6 (*WDR6*) was the top hit in this region associated with increased odds of AN – *Z*_TWAS_ = 7.44, *p* = 9.96 × 10^−14^ (GTEx cortex). This gene also surpassed Bonferroni correction in several other tissues, including the larger sample size PsychENCODE cortical samples, whole blood, nucleus accumbens, and caudate basal ganglia. The WD repeat protein family effects signal transduction (Li & Roberts, 2001), whilst *WDR6* is thought to influence cell cycle arrest (Xie, Wang, & Chen, 2007). Several other proximal genes to *WDR6* also survived Bonferroni correction in multiple tissues, such as, *CCDC71*, *NCKIPSD*, and *MST1R*. *O*-6-Methylguanine-DNA Methyltransferase (*MGMT*) was the most significant gene outside of chromosome 3 (*Z*_TWAS_ = −5.33, *p* = 9.88 × 10^−8^ – caudate basal ganglia), followed by *CDK11B* on chromosome 1, and the long non-coding RNA (lncRNA) *LINC00324* on chromosome 17. Previous analysis, differing in methodology and samples, found an association between *SUOX* expression and AN which was not replicated in the present study (Baird et al., 2021; Chatzinakos et al., 2020). Using a more lenient FDR approach for multiple-testing correction revealed more association signals (273 signals, 104 unique genes, online Supplementary Table S1). Additionally, we replicated 18 of the 36 significant associations from a previously published AN PrediXcan analysis, including *WDR6* and their most significant association *DALRD3*. We posit that any signals not replicated would be a function of FUSION only using *cis*-heritable genes, differences in GReX construction between the methods, and our study only including brain and blood tissues.

We then sought to extend the power for gene discovery, as well as supporting signals uncovered by the TWAS, through integration of models of genetically regulated protein expression (PWAS) and alternative splicing (spliceWAS, online Supplementary Tables S2, S3). In other words, these analyses considered the association of genetically predicted protein expression and alternative splicing with AN rather than mRNA expression. There were 2300 protein expression SNP weight sets in total available to test across the DLPFC and blood cohorts in which the imputed expression models were trained. Two proteins survived Bonferroni correction in the PWAS, and we uncovered four signals that were significant after applying a less stringent FDR based cut-off (FDR < 0.05). The strongest protein association, MHC class I chain-related gene B (MICB) (*Z*_PWAS_ = 4.53, *p* = 5.75 × 10^−6^, plasma) was correlated with increased odds risk of AN, however, due to the extensive LD of the MHC region it is difficult to apply approaches such as PWAS, and thus, this signal should be treated cautiously. The densely associated region on chromosome 3 harboured the next most significant protein expression signal, with decreased predicted expression of Glutathione peroxidase 1 (GPX1) associated with AN in the DLPFC (*Z*_PWAS_ = −4.41), mirroring its negative TWAS test statistic in the hypothalamus for mRNA expression (*Z*_TWAS_ = −4.51). Thereafter, the remaining two proteins surviving correction were each on different chromosomes (*FGF23,* chromosome 12 and *CTNND1,* chromosome 11). The spliceWAS yielded fourteen transcripts that survived Bonferroni correction from the 7708 transcripts tested relating to 3291 total genes. Once more the AN association dense region on chromosome 3 implicated already by GWAS, TWAS, and PWAS, yielded the most significant signal, with seven transcripts of Ariadne RBR E3 Ubiquitin Protein Ligase 2 (*ARIH2*) significantly associated. Interestingly, there were mixed directions of effect amongst the different isoforms as increased predicted abundance of three splice variants were associated with AN, whilst the converse was true for the remaining four. None of these isoforms correspond to the canonical transcript; chr3:48 918 821:48 967 151 (Howe et al., 2020). In general, the *ARIH2* gene is postulated to regulate ubiquitination (Kelsall et al., 2013; Marteijn et al., 2005) and post-transcriptionally modifies *NLRP3* to reduce inflammatory activity (Kawashima et al., 2017), however, the functional specificity of particular splicing isoforms is less well characterised. This gene was also not significant in the TWAS, despite having a well-powered model of imputed expression in the PsychENCODE cortical samples. The only remaining transcripts surviving Bonferroni correction not proximally located to the chromosome 3 region were two isoforms of GPR75-ASB3 Readthrough (*GPR75-ASB3*). Decreased predicted abundance of these two isoforms was associated with AN.

### Conditional analyses and probabilistic finemapping further refine AN association signals

Co-regulation and LD between genes can confound TWAS signals and lead to spurious associations for non-causal genes. Conditional analysis and finemapping was performed to distinguish genes with increased evidence of exerting an independent causal effect on AN. In other words, we tested whether there was statistical evidence to support each of our identified association signals as directly relevant for the disorder. Conditional analysis (Gusev et al., 2016) estimates the residual independent association of TWAS signals after controlling for the predicted expression of nearby significant genes. Benjamini-Hochberg significant TWAS genes were subjected to conditional analysis to predict which genes accounted for the localised signal. From 313 significant TWAS signals, 97 genes had a conditionally independent association (*p*_Joint_ < 0.05) with AN as indicated by their nominally significant joint *p* value (online Supplementary Table S4). The gene most significantly associated with AN, *WDR6* (*Z*_conditional−TWAS_ = 7.4, *p* = 1 × 10^−13^, Cortex) maintained an independent association after conditioning on the 77 other TWAS significant gene models (21 unique genes) from chromosome 3, three of which are also conditionally independent (*CTNNB1*, *GOLIM4*, *STX19*).

Finemapping through the FOCUS method (Mancuso et al., 2019) is a Bayesian statistical method designed to isolate subsets of genes more likely to contain causal genes based on a prior expectation of the number of causal genes we expect. We applied this approach to all suggestively significant regions in the AN GWAS (*p*_GWAS_ < 1 × 10^−5^) to derive 90% credible sets. Credible sets were removed if they contained the null model that is included by FOCUS to account for missing causal mechanisms like genes without a suitable GReX model. Across either mRNA, protein or mRNA alternative splicing weights, there were 116 genes that were prioritised in a credible set (online Supplementary Table S5). Of these, eight genes (Table 1) had moderate (*PIP* > 0.4) evidence of a causal effect on AN, while five genes demonstrated strong evidence (*PIP* > 0.8). The strongest evidence of a causal relationship was observed for Neurexophilin And PC-Esterase Domain Family Member 1 (*NXPE1*) (*PIP* = 1, testis). Surprisingly, *NXPE1* was indicated for the testis (GTEx), whilst it was also highly expressed in the colon (Aguet et al., 2017). The other four genes with strong evidence of a potential causal effect on AN were as follows: *WDR6* (*PIP* = 0.997, DLPFC), *PRKAR2A* (*PIP* = 0.814, GTEx artery tibial), *PROS1* (*PIP* = 0.895, GTEx cortex) and the non-coding RNA *RP13-238F13.5* (*PIP* = 0.971, GTEx spinal cord cervical c-1). *WDR6* and *MST1* were the only genes found to be both conditionally independent with at least moderate finemap evidence of a causal relationship (Table 1). Macrophage Stimulating 1 (*MST1*) mediates cell division and apoptosis (Wang et al., 2020; Zhang et al., 2019) and is predicted to increase risk of AN (*Z*_TWAS_ = 4.85, *p* = 1.2 × 10^−6^, GTEx Hypothalamus). However, finemapping of the MST1 PWAS indicates evidence of a protective relationship with AN (*Z*_finemap_ = −4.63, PIP = 0.457, blood plasma protein). This discordant direction between mRNA and protein requires further investigation to refine its biological salience. The power of finemapping to identify causal genes increases when more GReX expression models are included, and thus, tissues were used for finemapping that were not subjected to the marginal TWAS, since removing these would decrease finemapping power. There are three genes (*RP13- 28F13.5*, *NXPE1* and *PRKAR2A*) without TWAS blood and brain associations. One of these; *PRKAR2A*, exists in a credible set with four other genes (the same set as the finemapped *TREX1*), three of which are associated within brain regions.

**Table 1.** Finemapped associations

Molecular name	Tissue (finemapped model)	Ref name	*Z*_finemap_	PIP	*Z*_TWAS_ (P) -tissue	*Z*_PWAS_ (P) -tissue	Region	Credible set size (brain/ default)
*MST1*	Blood plasma protein	GTEx	−4.63	0.457	4.85 (1.2 × 10^−6^)- hypothalamus – Conditionally independent	−3.4787 (5.04 × 10^−4^) -plasma	3: 49 317 941–3: 51 830 833	NA/1
*WDR6*	Brain dorsolateral prefrontal cortex	PsychENCODE	6.46	0.997	7.44 (9.96 × 10^−14^)- cortex – Conditionally independent	NA	3: 49 317 338–3: 51 828 635	1/1
*RP13-238F13.5*	Brain spinal cord cervical c-1	GTEx	5.01	0.97	NA	NA	10: 125 919 795–10: 127 999 424	1/1
*NXPE1*	Testis	GTEx	6.02	1	NA	NA	11: 114 833 710–11: 116 383 064	1/1
*C3orf62*	Brain dorsolateral prefrontal cortex	CommonMind Consortium	−7.19	0.423	−7.13 (9.71 × 10^−13^)- cortex	NA	3: 47 777 774–3: 49 313 978	4/384
*TREX1*	Brain cortex	GTEx	−6.95	0.449	−3.2575 (1.1 × 10^−3^) – Nucleus accumbens basal ganglia	−2.1349 (0.032) -DLPFC	3: 49 317 338–3: 51 815 687	5/476
*PROS1*	Brain cortex	GTEx	5.21	0.895	0.07 (0.94) -whole blood	NA	3: 94 256 654–3: 95 306 175	2/3
*PRKAR2A*	Artery tibial	GTEx	−6.95	0.814	NA	NA	3: 49 317 338–3: 51 815 687	5/476

Finemapped genes with moderate or strong evidence of a causal effect on AN (PIP > 0.4). Tissue indicates which tissue the expression weights were derived from, Ref name indicates the group/consortium responsible for eQTL generation, *Z*_finemap_ indicates the estimated *Z* score association, PIP indicates the posterior inclusion probability and region indicates the GRCh37 genomic region the credible set was derived from. Credible set size indicates the number of genes in the credible set when prioritising brain or any tissue. All genes are in the credible set. *Z*_TWAS_ (P) -tissue and *Z*_PWAS_ (P) -tissue indicates the TWAS and PWAS *Z* score and *p* value associated with the most significant tissue for each association. No spliceWAS associations are available for these eight genes.

### Functional interrogation of prioritised AN risk genes suggests a role for immune function

Pathway analysis was performed on the conditionally independent (*N* = 97) and finemapped credible set of genes (*N*=8) using g:Profiler (Ensembl 103, Ensembl Genomes 50). We focused on pathways that were enriched for either of these input sets of genes that survived FDR correction (FDR < 0.05) and had at least three intersecting genes with the input list. Firstly, the conditionally independent genes were overrepresented amongst pathways related to the *presynaptic active zone* (overlapping genes = *STX19*, *CTNND1*, *CTBP2*, *CTNNB1*) (online Supplementary Table S6). While the genes prioritised by finemapping were overrepresented in the *regulation of immune system process* (overlapping genes = *MST1*, *TREX1*, *PRKAR2A*, *PROS1*) (online Supplementary Table S7). We note that as there were only eight genes that survived our finemapping pipeline that pathway analyses using this set of genes is somewhat underpowered.

## Discussion

We integrated genome wide association signals for AN with genetically regulated gene expression to identify novel risk genes and biological insights into the disorder. Critically, through the application of methods such as conditional analysis and finemapping, we refined a smaller set of genes with greater confidence of true association that can be subjected to future follow-up study. Leveraging proteomic and alternative splicing data also revealed association signals that were not seen using mRNA data alone. While previous studies have uncovered potential AN risk genes, our use of statistical finemapping and conditional analysis allowed us to prioritise confident associations such as *WDR6* that more likely exert a causal effect. Upregulation of *WDR6* exhibited a plausible causal relationship with AN across all methods with available data (TWAS, conditional analysis, finemapping). While the surrounding region is rich with genes predicted to have an association, a conditionally significant TWAS signal along with the finemapping evidence suggests that *WDR6* is a causal gene at this locus, however, further analyses such as SNP based finemapping and *in silico* prediction of variants in this locus are warranted to confirm this finding given genetic influence on disease may not directly be mediated by *cis*-acting expression. *WDR6* is a conserved repeat region expressed ubiquitously during human development that may function as a restriction factor to inhibit virus replication and a protein complex assembly platform (Li et al., 2000; Sivan, Ormanoglu, Buehler, Martin, & Moss, 2015; Smith, 2008). Previous work in a rodent model suggests *WDR6* may modulate insulin signalling in the hypothalamus (Chiba et al., 2009), which is interesting given that we observed evidence to suggest fasting insulin level exerts a protective effect against AN risk (Adams et al., 2021).

Mounting evidence suggests a relationship may exist between immune function and AN (Dalton et al., 2018a; Reay et al., 2022). Starvation can lead to inflammation; however, there remains differences between the presentation of immune dysregulation during malnutrition and AN, suggesting an underlying relationship may exist beyond the pathology associated with malnutrition (Gibson & Mehler, 2019). Interestingly, four of the eight finemapped genes were members in the *immune system processes* gene-set, which was a statistically significant overrepresentation. This is supportive of previous observations suggesting that immune system dysregulation may contribute to AN onset and maintenance (Gibson & Mehler, 2019). Furthermore, the strongest protein PWAS signal MICB, which implicates the MHC region, and the lead mRNA isoform gene *ARIH2* were also present within the immune system processes pathway. However, the overrepresentation in this instance was not significant like the finemapped genes. The identification of MICB in the PWAS necessitates further analysis of the MHC region in AN, such as the role of specific human leucocyte antigen types. Finemapped genes; *MST1* (Chanda et al., 2016; Lu, Zhao, & Liu, 2020), *PRKAR2A* (Kong et al., 2016) and *WDR6* (Lv, 2022) have previously been linked to inflammatory processes. Additionally, AN is also observationally associated with a pro-inflammatory state which includes increased levels of cytokines such as tumour necrosis factor and interleukins 1 and 6 (Caso et al., 2020; Dalton et al., 2018b; Gibson & Mehler, 2019). These studies are likely confounded by factors like reverse causality that complicates their interpretation, whilst there is evidence suggesting that some protein expression differences in severe AN may disappear after rehabilitation (Nilsson et al., 2020). Recent genetic studies suggest a protective effect of C-reactive protein (CRP) on AN, which may relate to infection susceptibility given that CRP is not simply a marker of inflammation (Reay et al., 2022; Tylee et al., 2018), as is often characterised, and directly participates in processes like phagocytosis (Dalton et al., 2018a; Del Giudice & Gangestad, 2018). Our data suggests that genes with immune related functions are involved in the pathogenesis of AN. Further work is now needed to refine whether the immune system is a plausible target for AN that could lead to new treatments. Pathway analysis performed on prioritised genes support these data that genetic risk for AN may exert a functional role in the immune system. The mechanistic action of the immune system in AN pathophysiology remains still largely uncharacterised, however, further experimental exploration of the specific genes prioritised in this study may reveal clinically relevant insights.

Whilst TWAS, SpliceWAS and PWAS provide a mechanistic framework for the associative evidence between genes and disease, there is also significant confounding by co-regulation derived correlation. TWAS associations can also be confounded by linkage disequilibrium which can bias SNP effect estimates for both expression weights and disease associations (Wainberg et al., 2019). Performing TWAS is also limited to some extent by the sample size of GReX data from different tissues and that some genes are not expressed and are therefore ‘missing’ from a relevant gene set. Incompleteness of gene expression data diminishes the effectiveness of finemapping, null models within the credible set could be better linked to potentially causal genes which would allow for the identification of other causal genes. Our understanding of the genetic architecture of AN is also far from complete, both in terms of common variants and effects mediated through rare or structural variants. Future larger-scale AN GWAS planned will help to consolidate the strength and replicability of these findings. Previous work in power analysis for TWAS approaches have suggested that both expression and trait heritability/sample size influence discovery power. The liability scale SNP heritability of AN even at current sample sizes is larger than several other psychiatric disorders, supporting that post-GWAS analyses can be deployed despite the above limitations. Moreover, expression-based approaches would be greatly improved by access to cell-type specific genetic models of expression. In terms of bulk-tissue panels, the PsychENCODE cortical dataset is well-powered and captures genetic effects on numerous genes, however, this is not the case for other brain regions and tissues in GTEx where sample size is much smaller. Another limitation of the eQTL association data used in this analysis is that it captures steady-state expression levels which cannot directly distinguish between decay rates of mRNA and transcriptional variance (Pai et al., 2012). Furthermore, these analyses were performed in exclusively European ancestries, and as more diverse non-European samples and trans-ancestry GWAS become available, this is likely to improve TWAS and finemapping studies (Aguet et al., 2017; Pai et al., 2012; Veturi & Ritchie, 2018; Watson et al., 2019). Finally, the existing AN GWAS has predominately female composition which somewhat restricts the identification of sex specific causal risk/protective factors.

In conclusion, our study highlighted novel genes, proteins and mRNA isoforms predicted to affect AN risk. We provide strong evidence suggesting that *WDR6* and several genes related to immune system function contribute to the pathogenesis of the disorder. Further research is warranted to establish these mechanisms and determine their potential as targets for treatment.

## Data Availability

All data in this study are publicly available. GWAS summary statistics for AN are available to download from the Psychiatric Genomics Consortium (https://www.med.unc.edu/pgc/download-results/). The TWAS and spliceWAS SNP weights are available from (http://gusevlab.org/projects/fusion/), whilst the PWAS SNP weights can be obtained from (http://nilanjanchatterjeelab.org/pwas/) and (https://www.synapse.org/#!Synapse:syn23627957) for plasma and brain, respectively. Code related to this study can be found at the following link: https://github.com/DanielleMAdams/Anorexia_nervosa_associated_genes_manuscript

## Supplementary material

For supplementary material accompanying this paper visit https://doi.org/10.1017/S0033291723000235. click here to view supplementary material
